# Correlation Between Conditions of Polyaniline Interlayer Formation and the Structure and Performance of Thin-Film Composite Membranes for Nanofiltration Prepared via Interfacial Polymerization

**DOI:** 10.3390/polym17091199

**Published:** 2025-04-28

**Authors:** Katsiaryna S. Burts, Tatiana V. Plisko, Anastasia V. Penkova, Bingbing Yuan, Sergey S. Ermakov, Alexandr V. Bildyukevich

**Affiliations:** 1 Department of Analytical Chemistry, Institute of Chemistry, St. Petersburg State University, 7/9 Universitetskaya nab., 199034 St. Petersburg, Russia or e.burt@spbu.ru (K.S.B.); a.penkova@spbu.ru (A.V.P.); s.ermakov@spbu.ru (S.S.E.); 2 Key Laboratory of Green Chemical Media and Reactions Ministry of Education, School of Chemistry and Chemical Engineering, Henan Normal University, Xinxiang 453007, China; yuanbingbing@htu.edu.cn; 3 Institute of Physical Organic Chemistry, National Academy of Sciences of Belarus, Surganov str., 13, 220072 Minsk, Belarus; uf@ifoch.bas-net.by

**Keywords:** nanofiltration, thin-film composite membrane, intermediate layer, polyaniline, interfacial polymerization

## Abstract

Correlations between conditions of the polyaniline (PANI) interlayer formation on the surface of a polysulfone (PSF) porous membrane substrate and the structure and performance of thin-film composite (TFC) membranes for nanofiltration with a polyamide (PA) selective layer prepared via interfacial polymerization (IP) were studied. It was shown that application of the PANI layer significantly enhanced hydrophilicity (the water contact angle decreased from 55 ± 2° down to 26–49 ± 2°), decreased pore size and porosity, and increased the surface roughness of the selective layer surface of porous PSF/PANI membrane substrates due to the formation of bigger PANI globules, which affect the formation of the PA layer of TFC membranes via IP. It was shown that the application of the PANI intermediate layer yielded the formation of a thinner PA selective layer, a decline in surface roughness, and an increase in hydrophilicity (the water contact angle declined from 28 to <10°) and crosslinking degree of the selective layer of TFC NF membranes. The developed approach allows us to enhance the water permeation up to 45–64 L·m^−2^·h^−1^ at ΔP = 0.5 MPa and improve membrane selectivity (rejection coefficient of MgSO_4_—>99.99%; LiCl—5–25%; sulfadimetoxine—80–95%) and also ensure enhanced long-term operational stability of TFC nanofiltration membranes with a PANI interlayer. Moreover, Mg^2+^/Li^+^ separation factor values were found to increase to 37 and 58 for PANI-modified membranes compared to 9 and 8 for the reference NF-PSF membranes.

## 1. Introduction

Thin-film composite (TFC) membranes with a polyamide (PA) selective layer have become a key focus within the field of nanofiltration membrane preparation [1]. Nanofiltration (NF) is a pressure-driven process that is usually applied to separate divalent/multivalent ions, salts, and antibiotics in water treatment [2,3]. Nanofiltration is characterized by a number of advantages, such as low energy consumption, high purification efficiency, flexibility in application, and lack of secondary pollution [2]. The PA selective layer is usually formed on the surface of a porous ultrafiltration (UF) or microfiltration (MF) membrane substrate via the interfacial polymerization (IP) reaction between multifunctional amine monomers in the aqueous solution and multifunctional acyl chloride in the organic phase [4]. The commonly used multifunctional amines are m-phenylenediamine, p-phenylenediamine, and piperazine (PIP) [5,6,7]. In addition to the mentioned amine monomers, polyethyleneimine (PEI) is also used as an amine component to obtain a PA selective layer [8]. The commonly used multifunctional acyl chloride monomers are trimesoyl chloride (1,3,5-benzenetricarbonyl trichloride, TMC) and isophthaloyl chloride (IPC) [9,10]. The uncontrolled diffusion of amine monomers to an interphase boundary poses a significant challenge in achieving precise control over the morphology and structure of the resulting PA layer. This lack of control often results in the underperformance of nanofiltration membranes compared to their expected efficiency [11].

Due to the trade-off between the permeability and selectivity of nanofiltration membranes, optimization of the PA layer is still relevant (varying the nature and concentrations of the reagents in IP reaction, the duration of soaking in solutions, and the drying temperature) [12,13,14,15]. PIP and TMC are more typically used for the preparation of PA selective layers for NF membranes [1,16,17,18].

Moreover, there are some other techniques to break the permeability–selectivity trade-off: hydrophilization of the membrane substrate, formation of the intermediate layer, introduction of different fillers (nanoparticles, metal–organic frameworks, and surfactants) to the PA selective layer [19,20,21,22,23,24]. The formation of the intermediate layer was found to be the prospective technique for improving the nanofiltration membrane’s performance. The interlayer can enhance hydrophilicity, reduce the thickness of the PA layer, and create nanostructures by controlling the IP reaction [25]. It was reported that the intermediate layers were formed from TiO_2_ [26], MoS_2_ [27,28], nanocellulose [29,30], carbon nanotubes [31,32,33], metal–organic frameworks (MOFs) and covalent organic frameworks (COFs) [34,35,36,37,38], polymer/MOF (COF) composites [39,40,41,42], polydopamine (PDA) [20,43,44], poly (sodium 4-styrenesulfonate)/PEI [45], PEI [46], sodium alginate [47], polyphenols [48,49,50], etc.

Unlike inorganic nanomaterials, polymeric coatings can establish robust adhesion forces of a PA selective layer with the surface of a membrane substrate through electrostatic interactions, hydrogen bonds, or Van der Waals forces. These interactions contribute to enhancing the structural stability of the TFC membrane [51].

Polydopamine (PDA) is widely used for preparation of the intermediate layer of nanofiltration membranes [20,43,52,53,54,55]. Al-Nahari et al. [20] obtained nanofiltration membranes by deposition of a PDA intermediate layer on the surface of a PSF ultrafiltration membrane with a molecular weight cut-off (MWCO) of 70 kDa, followed by the formation of a PA selective layer from PIP and TMC via IP. It was found that the deposition of the PDA intermediate layer resulted in a remarkably thin PA layer of 8 nm and a low average surface roughness parameter (R_a_) of 3.2 nm. The resulting PDA-TFC nanofiltration membranes exhibited high performance, achieving a Na_2_SO_4_ rejection coefficient of 99.98%, with a pure water permeance of 34 L·m^−2^·h^−1^·bar^−1^ [20].

Yang et al. [43] prepared nanofiltration PANI/PDA/PA membranes similar to membranes developed by Al-Nahari et al. [20] but with a denser selective layer. It was revealed [43] that PANI/PDA/PA nanofiltration membranes were characterized by lower water permeance (15 L·m^−2^·h^−1^·bar^−1^) and a Na_2_SO_4_ rejection coefficient of 90–95%, depending on the deposition time of the intermediate layer compared to PDA/PA membranes [20].

A literature analysis revealed that there are a lot of studies dedicated to the development of nanofiltration membranes with a modified PDA intermediate layer. Thus, the PDA intermediate layer was formed with the addition of PEI [52,53], graphene oxide (GO) [54], UiO-66 carbon nanotubes [42], and MXene [55]. It was shown that these additives significantly increased the surface hydrophilicity and enhanced the water permeance without a significant decline in selectivity.

According to the literature analysis, it was revealed that different polymers were used for the formation of the intermediate layer: poly (sodium 4-styrenesulfonate) (PSS)/polyethyleneimine (PEI) with the polyelectrolyte complex formation [45], sodium alginate [47], polyvinyl alcohol (PVA) [25], PVA/tannic acid [56], hyaluronic acid [57], PEI crosslinked with glutaraldehyde [46], and glutaraldehyde crosslinked chitosan [58].

It was found that nanofiltration membranes with an intermediate layer based on the above-mentioned polymers except the PEI/GA interlayer [46] resulted in the hydrophilization of the selective layer. Regarding the membrane performance, the formation of a polymer intermediate layer resulted in enhanced water permeance, excluding membranes with interlayers based on hyaluronic acid [57] and chitosan crosslinked by glutaraldehyde [58]. However, it was revealed that the mentioned membranes with a polymer intermediate layer possessed an advanced selectivity compared to the TFC membranes for nanofiltration without an interlayer.

Zha et al. applied a PANI interlayer by an in situ polymerization method on the surface of a hydrolyzed polyacrylonitrile (HPAN) ultrafiltration membrane (MWCO—50 kDa), followed by PA selective layer formation via IP between PEI and TMC [51]. The performance of the prepared membranes was investigated in organic-solvent nanofiltration. It was shown that the PA/PANI/HPAN membrane was characterized by an ethanol permeance of 3.1 L·m^−2^·h^−1^·bar^−1^ and a rejection coefficient for Eriochrome black T, Acid fuchsin, Congo red, Brilliant blue R, and Rose Bengal dyes of over 98%.

Guo et al. obtained sulfonated polyaniline (SPANI) nanofibers in order to apply them for intermediate layer formation on the surface of polyethersulfone (PES) microfiltration (MF) membranes with an effective pore size of 0.45 μm [59,60]. The selective PA layer was prepared via the traditional IP process using PIP and TMC solutions. It was found that the NF membrane with a SPANI intermediate layer was characterized by a water permeance of 29 L·m^−2^·h^−1^·bar^−1^ and a Na_2_SO_4_ rejection of 99% compared to the reference membrane with a water permeance of 12 L·m^−2^·h^−1^·bar^−1^ and a Na_2_SO_4_ rejection of 94% [59,60].

However, according to the literature analysis, no works devoted to the study of the effect of the application modes of the PANI layer via oxidative polymerization (concentration of aniline and duration of PANI intermediate layer deposition) on the formation of a PA selective layer via IP and the structure and performance of the resulting TFC nanofiltration membranes were reported. The aim of this research was to investigate the patterns of the formation of the PA selective layer via IP depending on the conditions of intermediate PANI layer formation. Moreover, the influence of the concentrations of reagents during IP on the structure and performance of TFC membranes for nanofiltration was studied. The developed membranes were characterized by scanning electron microscopy (SEM), atomic force microscopy (AFM), contact angle measurements, and X-ray photoelectron spectroscopy (XPS). Membrane performance was studied in ultra- and nanofiltration using polyvinylpyrrolidone (PVP K30, M_n_ = 40,000 g·mol^−1^), salts and antibiotic sulfadimetoxine, and mixtures of magnesium sulfate and lithium chloride as test substances. The novelty of the present work is that for the first time, the correlation between the conditions of the PANI interlayer formation on the surface of a polysulfone (PSF) ultrafiltration (UF) membrane substrate and the structure and performance of thin-film composite (TFC) membranes for nanofiltration with a polyamide (PA) selective layer prepared via interfacial polymerization (IP) were revealed.

## 2. Materials and Methods

### 2.1. Materials

Polysulfone Ultrason S 6010 (PSF, M_n_ = 55,000 g·mol^−1^) and N,N-dimethylacetamide (DMAc) were acquired from BASF (Ludwigshafen, Germany) and used for membrane substrate preparation. Polyvinylpyrrolidone (PVP K30, M_n_ = 40,000 g·mol^−1^), used as a pore-forming agent, was provided by Fluka (Munich, Germany). Aniline (ANI, 99.5% purity) was sourced from Sisco Research Laboratories Pvt. Ltd. (Mumbai, India). Concentrated hydrochloric acid (HCl) was purchased from AnalytComplect (Minsk, Belarus). Ammonium persulfate (APS, (NH_4_)_2_S_2_O_8_) was obtained from Vecton (St. Petersburg, Russia). Piperazine (PIP, 98% purity) and trimesoyl chloride (TMC, benzene-1,3,5-tricarbonyl chloride, 98% purity) were supplied by Sisco Research Laboratories Pvt. Ltd. (Mumbai, India) and Tokyo Chemical Industry Co., Ltd. (Tokyo, Japan), respectively.

Magnesium sulfate (MgSO_4_·7H_2_O, AnalytComplect, Minsk, Belarus), sodium chloride (NaCl, Belleshimkomplekt, Minsk, Belarus), calcium chloride (CaCl_2_, anhydrous, Belleshimkomplekt, Minsk, Belarus), magnesium chloride (MgCl_2_·6H_2_O, Serva, Heidelberg, Germany), sodium sulfate (Na_2_SO_4_·10H_2_O, AnalytComplect, Minsk, Belarus), lithium chloride (LiCl, Lenreactiv, St. Petersburg, Russia), and sulfadimetoxine (SDM, M = 310.33 g·mol^−1^, BLDpharm, Shanghai, China) were applied as test substances to investigate membrane performance in nanofiltration.

### 2.2. Methods

#### 2.2.1. Membrane Preparation

The polymer solutions were prepared in a glass flask under constant stirring using an IKA RW20 (IKA Werke GmbH & Co. KG, Staufen im Breisgau, Germany) overhead stirrer at 120 °C for 4–5 h. The resulting homogeneous solution was then left to stand overnight to remove air bubbles.

UF membranes were prepared via a non-solvent induced phase separation technique using a water coagulation bath at room temperature. Polymer solution was cast on the polyester nonwoven fabric by the casting knife with the gap of 250 µm. After polymer solution casting, the film was immersed in the water coagulation bath. Prepared UF membranes were left for one day to remove residual solvent and wash out the pore-forming agent.

The polyaniline (PANI) interlayer was formed by oxidative polymerization of ANI on the surface of the UF PSF membrane substrate at the temperature of 4–10 °C by physical adsorption from the aniline solution in 1 M HCl with ammonium persulfate as an initiator under constant stirring (Figure S1). The scheme of oxidative polymerization of aniline is presented in the Supporting Information (Figure S1). The PSF membrane was fixed upside down in such a way that only the selective layer surface contacted the solution, where ANI oxidative polymerization occurred. After ANI polymerization, the membrane was kept in water for 24 h to remove unreacted monomer from the membrane surface. The duration of PANI layer formation was 0.5 h or 1 h. The concentrations of ANI in the aqueous solution were 0.05, 0.1, and 0.3 wt.%. The codes of the prepared UF membranes with the PANI layer are presented in Table 1.

The polyamide (PA) selective layer was formed by interfacial polymerization (IP) via the reaction between PIP aqueous solutions and TMC solution in Nefras C2 (80/120, “Vershina” LLC, Vsevolozhsk, Russia). The membrane was kept in each solution for 10 s in such a way that only a selective layer contacted with the solutions of monomers. The excess PIP aqueous solution was gently removed by a rubber roller. After applying the PA layer, the membrane was dried for 10 min at 80 °C. The PIP concentration was varied from 1 to 4 wt.%, while the mass ratio PIP/TMC remained constant. (±)-Camphor-10-sulfonic acid (CSA, Maclin, Shanghai, China) was used as an additive to the PIP aqueous solution at a concentration similar to the PIP concentration. The codes of prepared TFC NF membranes are listed in Table 1.

#### 2.2.2. Membrane Characterization

##### Study of Membrane Structure by Scanning Electron Microscopy (SEM)

To investigate the membrane morphology, a scanning electron microscope Zeiss AURIGA Laser (Carl Zeiss AG, Oberkochen, Germany) was used. Membranes were immersed in 20 wt.% glycerol aqueous solution and dried in air before SEM studies. Impregnation of membranes in glycerol aqueous solution allows the membrane pore structure to be preserved upon drying. Membrane cleavages were prepared by submerging membrane samples in liquid nitrogen and fracturing perpendicular to the surface.

##### Study of Membrane Surface Structure by Atomic Force Microscopy (AFM)

The surface topography of the developed UF and NF membranes was studied by an NTMDT nTegra Maximus atomic force microscope with standard silicon cantilevers with a rigidity of 15 N·m^−1^ (NT-MDT Spectrum Instruments, Zelenograd, Russia) in tapping mode.

##### Fourier-Transform Infrared (FTIR) Spectroscopy

The composition of selective layers of PSF membrane substrates was analyzed by Fourier-transform infrared (FTIR) spectroscopy using a Nicolet Is50 spectrometer (Thermofisher Scientific, Waltham, MA, USA). Spectra were acquired in the range of 400–4000 cm^−1^ at 25 °C. Prior to analysis, all membrane samples were air-dried at ambient temperature for 5 days to ensure complete water evaporation.

##### X-Ray Photoelectron Spectroscopy (XPS)

X-ray photoelectron spectroscopy (XPS) measurements were conducted using an ESCALAB™ 250Xi spectrometer (Thermo Fisher Scientific, Waltham, MA, USA) equipped with a monochromatic Al-Kα X-ray source (650 μm spot size). Analyses were performed at a 90° take-off angle relative to the surface plane. Elemental composition was quantified from survey spectra (0–1350 eV) acquired with a 100 eV pass energy, a 0.5 eV step size, a 50 ms dwell time, and 5 scan accumulations. Atomic concentrations were derived by integrating peak intensities with Smart-type background subtraction. Five different surface locations were analyzed on each sample surface, and average values were calculated.

High-resolution spectra of C1s, O1s, and N1s core levels were recorded with a 20 eV pass energy, a 0.05 eV step resolution, a 50 m·s dwell time, and 50 accumulated scans. Spectral deconvolution was performed using Thermo Scientific™ Advantage software, applying a Smart-type background subtraction with constrained parameters: 1.5 eV full width at half maximum (FWHM) and 35% Gaussian–Lorentzian peak shape mixing.

The polyamide network architecture comprises two distinct structural domains:

- A crosslinked domain (m) formed when all three acyl chloride functionalities of TMC react with PIP amine groups;
- A linear domain (n) resulting from partial reaction where only two acyl chlorides participate in amide bond formation [32].

These structural motifs exhibit characteristic oxygen-to-nitrogen stoichiometric ratios:

A 3:3 O:N ratio for fully crosslinked segments (m);

A 4:2 O:N ratio for linear segments (n).

The degree of crosslinking (D) was quantitatively determined through systematic analysis using Equations (1) and (2), which account for these structural variations and stoichiometric relationships:

(1) $$\frac{O}{N}=\frac{3m+4n}{3m+2n},$$

(2) $$D=\frac{m}{m+n}\times 100\%=\frac{4N-2O}{N+O}\times 100\%.$$

In Equations (1) and (2), O (%) and N (%) indicate the respective ratios of O and N.

##### Zeta Potential of the TFC NF Membrane Delective Layer

Zeta potentials of the selective layers of TFC NF membranes were determined using an Anton Paar SurPass 3 (Austria, Graz) solid surface analyzer. The membrane samples were kept in MilliQ water for 24 h before tests. The sample holder for discs was used; the gap height between two discs of the membrane sample was set at 100–106 µm. Aqueous potassium chloride solution in MilliQ water with a concentration of 1 mmol·L^−1^ was used as an electrolyte. The zeta potential was measured in the pH range of 3.0 to 10.0, with steps of 0.5–0.7 pH units.

##### Contact Angle

The contact angles of UF and NF membranes with a PANI layer were analyzed by the attached bubble method using an LK-1 goniometer (RPC «Open Science Ltd.»). Contact angle measurements were conducted in a three-phase system comprising water, a membrane surface, and an air bubble. For each batch, measurements were performed on five membranes. The measurement error was within ±2°.

##### Preparation of the Solutions of Test Substances

To evaluate the performance of TFC NF membranes, aqueous solutions of salts MgSO_4_ (2 g·L^−1^), Na_2_SO_4_ (2 g·L^−1^), MgCl_2_ (2 g·L^−1^), CaCl_2_ (2 g·L^−1^), LiCl (1 g·L^−1^), NaCl (0.5 g·L^−1^), and SDM (20 mg·L^−1^) were prepared at room temperature by dissolving a predetermined amount of the test substance in distilled water with continuous stirring.

##### Membrane Performance

Membrane performance was assessed using a custom-designed filtration cell with a volume of 400 mL and a membrane area of 24.6 cm^2^. The separation efficiency of the reference UF PSF membrane substrate was evaluated by measuring the pure water flux (J_water_, L·m^−2^·h^−1^) and the rejection coefficients for polyvinylpyrrolidone (PVP K30, M_n_ = 40 kDa) (R, %). Specifically, the UF membrane was conditioned by filtering distilled water at 0.1 MPa for 30 min at room temperature while stirring at 250–300 rpm, after which the pure water flux was recorded. The rejection coefficients for PVP K-30 were measured 20 min after the filtration of a 0.3 wt% PVP K-30 aqueous solution.

To determine the membrane rejection coefficients, the concentration of PVP K-30 in the feed and permeate was determined using an ITR-2 interferometer (Zagorsk Optical-Mechanical Plant, Sergiyev Posad, Russia) with a cell length of 40 mm, 150-fold lens magnification, and an incandescent lamp as a light source. The interferometer LIR-2 determines the concentration of the solution via the difference between the refractive indices of the studied solution and a standard solution or liquid with a known refractive index. The principle of operation of the interferometer LIR-2 is based on double-slit diffraction phenomenon.

The membrane performance of TFC NF membranes was systematically evaluated through filtration experiments in stirring mode conducted at ambient temperature (25 ± 1 °C) under an applied pressure of 0.5 MPa. The investigation encompassed three feed solutions: distilled water, saline solutions, and antibiotic-containing aqueous systems. A stirring rate of 400 rpm was maintained throughout the experiments to minimize concentration polarization effects.

Prior to performance characterization, membranes underwent a stabilization protocol consisting of 1 h distilled water filtration to establish steady-state operation. Initial water permeability measurements were subsequently recorded. The feed solution was then replaced with the test solutions for rejection studies.

Comprehensive performance assessment included:

- Water permeation flux determination;
- Quantitative evaluation of salt rejection efficiency;
- Antibiotic removal capacity analysis.

All performance characteristics were measured following a 2 h equilibration period to ensure system stability and reproducible results.

The membrane rejection coefficient was determined by measuring the concentrations of salts in both the permeate and feed solution using an ITR-2 interferometer. The rejection coefficients for the antibiotic SDM were assessed with a UV/Visible Spectrophotometer SP-8001 (Metertech Inc., Taipei, Taiwan) at a wavelength of 280 nm.

The membrane’s ion rejection was evaluated using a mixed aqueous solution containing LiCl and MgSO_4_ with the following characteristics:

- Total dissolved salt concentration: 5 g·L^−1^;
- Mg^2+^/Li^+^ mass ratio: 18:1.

Feed and permeate compositions were quantitatively analyzed using inductively coupled plasma atomic emission spectroscopy (ICP-AES, Vista PRO, Varian, Palo Alto, CA, USA). The separation factor (S_Mg_^2+^_,Li_^+^), representing the membrane’s preferential selectivity between divalent and monovalent cations, was calculated using Equation (3):

(3) $$S{Mg}^{2+},{Li}^{+}=\frac{\left(C_{Mg2+}/C_{Li+}\right)f}{\left(C_{Mg2+}/C_{Li+}\right)p}=\frac{100-R_{Li+}}{100-R_{Mg2+}},$$

where C_Mg_^2+^ and C_Li+_ represent concentrations of Mg^2+^ and Li^+^ in feed solution (f) and permeate (p), respectively, while R_Li_^+^ and R_Mg_^2+^ denote the rejection coefficients of Li^+^ and Mg^2+^, respectively.

##### Pore Size Distribution of TFC NF Membranes

The mean effective pore size of the fabricated nanofiltration membranes was characterized using a neutral solute rejection method. Aqueous solutions containing polyethylene glycol (PEG) with molecular weights of 200, 300, 400, and 600 Da (each at 0.3 wt.% concentration) were employed as probe molecules for filtration experiments. The hydrodynamic Stokes radius (r_s_, nm) of each PEG molecule was calculated using Equation (4), enabling quantitative assessment of the membrane’s pore size distribution based on size-exclusion characteristics.

(4) $$r_{s}=16.74\times 10^{-3}\times M_{W}^{0.557}$$

The hydrodynamic radius of polyethylene glycol (PEG) molecules was determined using Equation (4). Membrane pore structure parameters, including mean effective pore diameter (μ_p_) and pore size distribution, were quantified following established protocols [61]. The analytical procedure involved the following steps:

- Construction of a semi-logarithmic probability plot of solute rejection versus Stokes radius;
- Determination of μ_p_ as the intercept at 50% rejection from the fitted curve;
- Calculation of the geometric standard deviation (σ_g_) from the ratio of solute radii at 84.13% and 50% rejection thresholds.

Under the assumption of negligible steric and hydrodynamic interactions between solute molecules and pore walls, the obtained μ_p_ and σ_g_ values correspond to the actual mean pore size (μ_s_) and size distribution (σ_g_), respectively. The pore size distribution was mathematically described by the probability density function (PDF) given in Equation (5):

(5) $$\frac{dR_{T}(r_{p})}{dr_{p}}=\frac{1}{r_{p}ln\sigma_{p}\sqrt{2\pi }}\exp\left(-\frac{{\left(lnr_{p}-ln\mu_{p}\right)}^{2}}{2{\left(ln\sigma_{p}\right)}^{2}}\right).$$

##### Determination of the Molecular Weight Cut-Off (MWCO) of TFC NF Membranes

The investigation of the molecular weight cut-off (MWCO) was conducted during the filtration of 0.3 wt. % aqueous solutions of PEG with different molecular weights (200, 300, 400, and 600 Da). To obtain MWCO values of TFC NF membranes, the rejection coefficients of tested PEGs were calculated, and the relationship between the rejection and PEG molecular weight was plotted. The MWCO was considered as a molecular weight at 90% rejection.

##### Study of TFC NF Membrane Chemical Stability

Chlorine resistance evaluation: The membrane was immersed in 2000 ppm NaClO solution for oxidation treatment, and the pH of NaClO was controlled at pH = 8 with hydrogen chloride and sodium hydroxide solutions. The NaClO solution was replaced every hour for 12 h to prevent any concentration change in the solution. Thereafter, the water permeation flux at ΔP = 0.5 MPa and MgSO_4_ rejection were measured.

Acid resistance evaluation: The membrane was immersed in 10 wt % H_2_SO_4_ acid solution and heated to 80 °C for 3 h. Thereafter, the water permeation flux at ΔP = 0.5 MPa and MgSO_4_ rejection were measured.

## 3. Results

### 3.1. Effect of PANI Layer Deposition at Different Aniline Concentrations During Oxidative Polymerization on the UF PSF Membrane Structure and Performance

**Figure 1:**
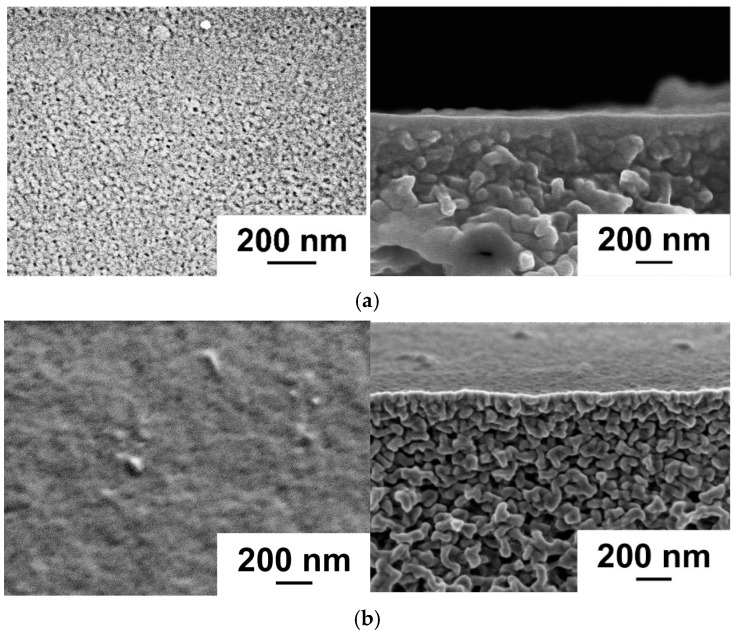
SEM microphotographs of the surface and cross-section of the selective layer of ultrafiltration PSF membrane substrate: (**a**) reference UF-PSF; (**b**) UF-PSF/PANI 0.1.

The structure of the reference PSF membrane substrate and PSF membranes with a deposited PANI layer was studied by SEM (Figure 1). It was revealed that the formation of a PANI layer on the surface of the UF-PSF reference membrane resulted in partial blocking of the pores of the selective layer of the membrane substrate and a reduction in the porosity of the selective layer (Figure 1). Moreover, PANI formations of irregular shape were observed on the selective layer surface of UF-PSF/PANI membranes. The PANI layer was undetectable via SEM of the membrane cross-section; therefore, it was impossible to determine the PANI layer thickness from SEM micrographs.

**Figure 2:**
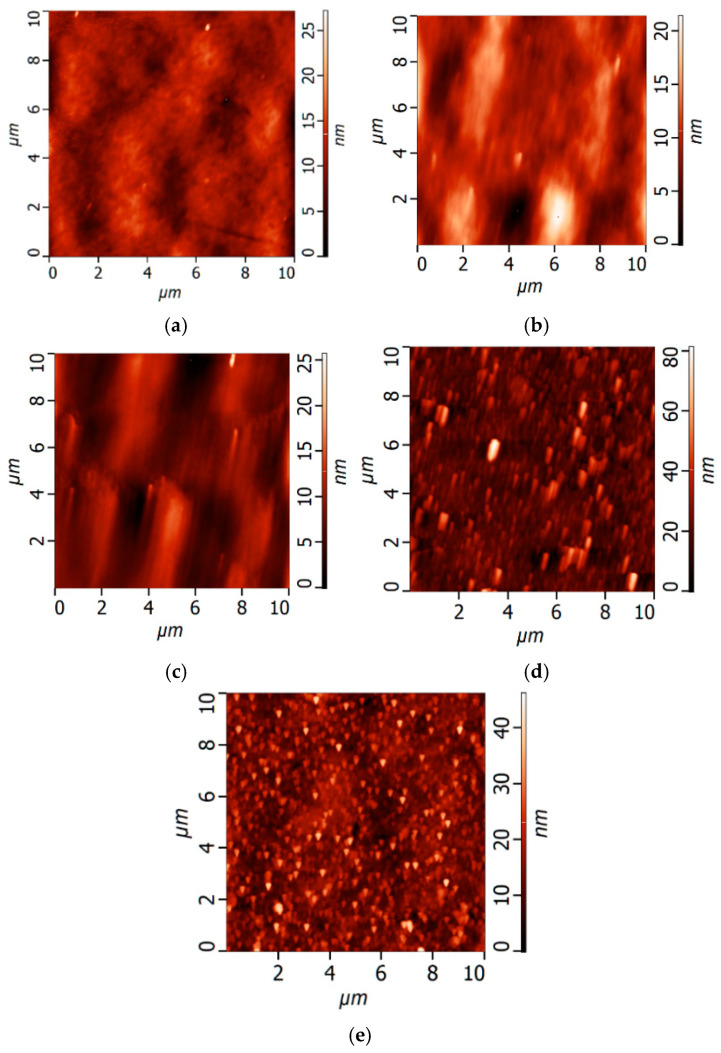
AFM microphotographs of UF PSF membranes with PANI layer: (**a**) reference UF-PSF; (**b**) UF- PSF/PANI 0.05; (**c**) UF- PSF/PANI 0.1; (**d**) UF-PSF/PANI 0.3, (**e**) UF- PSF/PANI 0.1–1.

The topography of the surface of UF membranes with a deposited PANI layer was investigated by atomic force microscopy (AFM) (Figure 2, Table 2). According to AFM investigations, the surface of the selective layer consisted of polymer nodules, similar to other membranes prepared by the NIPS method. It was demonstrated that the formation of a PANI layer on the selective layer surface of the PSF membrane led to an increase in the surface roughness parameters (average surface roughness (R_a_) and root-mean-squared surface roughness (R_q_)) (Figure 2, Table 2). This is due to the formation of PANI globules of irregular shape on the selective layer surface proved by SEM studies (Figure 1). Moreover, an increase in the concentration of monomers (ANI) in the oxidative polymerization process resulted in a significant rise in the roughness of the UF membrane surface due to the formation of bigger PANI globules (Table 2). It was found that increasing the time of PANI layer deposition from 0.5 h up to 1 h at an ANI concentration of 0.1 yields an increase in surface roughness parameters from R_a_ = 2.19 nm and R_q_ = 2.74 nm for the UF-PSF/PANI 0.1 membrane to R_a_ = 3.94 nm and R_q_ = 5.24 nm for UF-PSF/PANI 0.1–1. Visually, a darker membrane surface was formed when the PANI layer was applied for 1 h compared to 0.5 h.

The hydrophilic–hydrophobic balance of the selective layer surface of UF PSF/PANI membranes was studied by the determination of the water contact angle (Table 2). It was found that an increase in the concentration of ANI during the formation of the PANI layer on the surface caused a significant enhancement of surface hydrophilicity, confirmed by the decrease in water contact angle (Table 2). The water contact angle of UF PSF/PANI membranes was shown to decline down to 26–49°, compared to 55° for the initial UF PSF membrane, which indicated significant surface hydrophilization (Table 2). It should be mentioned that the initial membrane substrate UF-PSF had a lower water contact angle than pristine PSF (77–80°), attributed to the addition of very hydrophilic PVP K30 to the casting PSF solution.

The formation of a PANI layer on the surface of UF PSF membranes was proved by FTIR studies (Figure S2). It was found that vibrations of the main membrane material PSF (1150, 1586, 1487, 1150, 1243, and 2960 cm^−1^) predominate in all spectra (Figure S2). The peak at 1650 cm^−1^ is due to the vibrations of the carbonyl group of PVP K-30. The presence of polyaniline on the membrane surface is proved by the appearance of vibrations of the secondary amine group at 3293 cm^−1^ (Figure S2).

Moreover, it was shown that rise in the duration of PANI layer deposition from 0.5 h up to 1 h at an ANI concentration of 0.1 results in a decline in water contact angle down to 30 ± 2°, which demonstrated the formation of a more hydrophilic selective layer surface.

**Figure 3:**
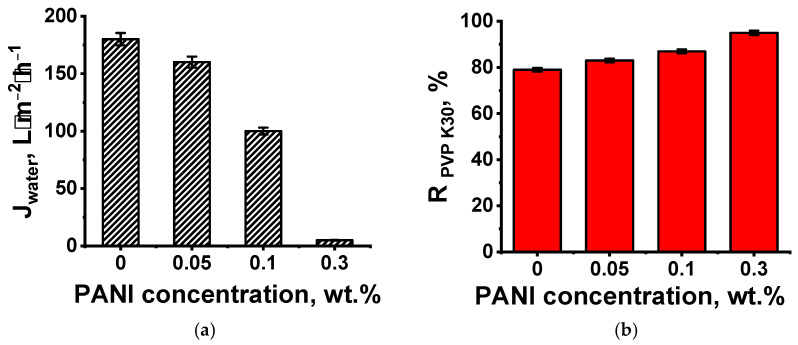
Performance of UF membranes with a PANI layer obtained during 0.5 h of application depending on ANI concentration during oxidative polymerization: (**a**) pure water flux; (**b**) rejection coefficient of PVP K30.

UF PSF/PANI membranes were examined during water and PVP K30 aqueous solution ultrafiltration (Figure 3).

It was shown that the formation of a PANI layer on the surface of a PSF porous membrane resulted in a decrease in pure water flux (Figure 3). An increase in ANI concentration during oxidative polymerization led to a significant decline in membrane permeability due to the decrease in porosity and pore size of the selective layer, since pores are blocked by the PANI layer. It was also found that an increase in ANI concentration up to 0.3 wt.% during oxidative polymerization on the surface of the UF PSF membrane support yielded a crucial decline in water permeability down to 5 L·m^−2^·h^−1^ at ΔP = 0.1 MPa (Figure 3a). This is likely due to the increase in the PANI layer thickness and decrease in its pore size and porosity. It was revealed that membranes with a layer applied from 0.05 and 0.1 wt.% ANI solution are characterized by a pure water flux of 160 and 100 L·m^−2^·h^−1^ at ΔP = 0.1 MPa, respectively. An increase in the concentration of ANI in the aqueous solution resulted in a decline in the pore size of the selective layer, which caused a rise in membrane rejection—the PVP K30 rejection coefficient rose from 79 to 83–95% (Figure 3b). It was shown that the increase in the deposition time of the PANI layer from 0.5 h up to 1 h at an ANI concentration of 0.1 wt.% yields a decrease in pure water flux from 100 L·m^−2^·h^−1^ at ΔP = 0.1 MPa down to 84 L·m^−2^·h^−1^ at ΔP = 0.1 MPa. The rejection of PVP K30 remains at the same level of 87%. This is due to the formation of a thicker PANI layer with lower porosity. The obtained results showed that UF-PSF/PANI 0.05 and UF-PSF/PANI 0.10 membranes were characterized by a lower molecular weight cut-off (MWCO)—approximately 20 kDa, compared to the reference UF PSF membrane with an MWCO of approximately 50 kDa.

Thus, it was found that deposition of a PANI layer via oxidative polymerization on the surface of a PSF ultrafiltration membrane yields partial pore blocking, an increase in surface roughness, and hydrophilization of the membrane selective layer. When a higher ANI concentration and longer time of PANI layer formation are applied, a rougher and more hydrophilic selective layer with smaller pores is formed. It is expected that these substantial changes in structure, porosity, and hydrophilic–hydrophobic properties of the membrane selective layer will affect the PA layer formation via IP during TFC membrane preparation.

### 3.2. Effect of PANI Interlayer Formation on the Structure and Performance of TFC Membranes for Nanofiltration Prepared via IP

TFC nanofiltration membranes with a PANI interlayer were obtained via IP. The PANI interlayer was applied for 0.5 h or 1 h using concentrations of 0.05 wt.%, 0.1 wt.%, 0.3 wt.%, and 0.5 wt.% of the monomer ANI in the aqueous solution during oxidative polymerization. The effect of the modes of PANI interlayer formation on the structure and performance of TFC membranes for nanofiltration was revealed. Moreover, the influence of PIP and TMC concentrations during IP on the structure and performance of TFC NF membranes was studied.

#### 3.2.1. The Effect of Time of PANI Interlayer Deposition on the Structure and Performance of TFC NF Membranes

**Figure 4:**
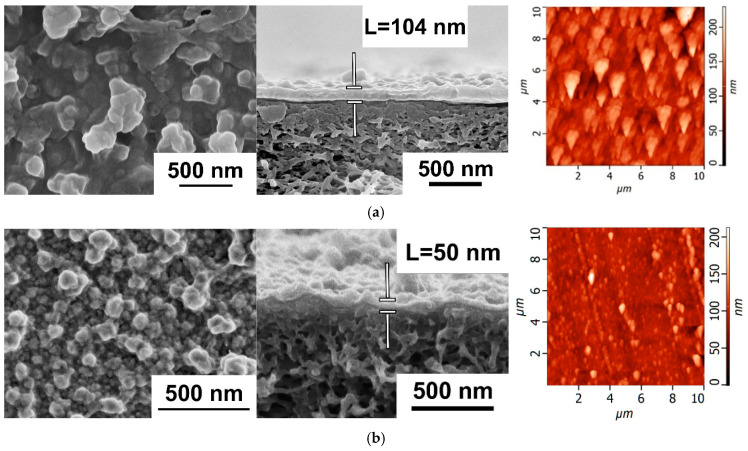

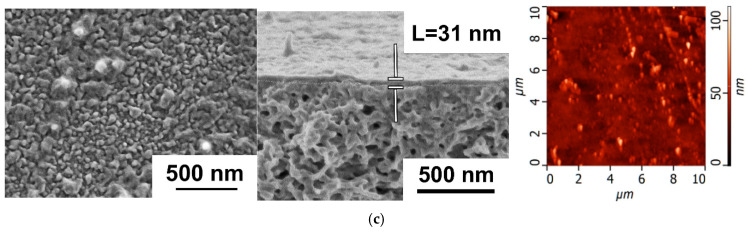
SEM and AFM microphotographs of the selective layer surface and cross-sections of TFC NF membranes depending on the duration of PANI interlayer formation: (**a**) NF-PSF–4; (**b**) NF-PSF/PANI 0.1–0.5–4; (**c**) NF-PSF/PANI 0.1–1–4. Figure 4a was adapted with permission from Ref. [62]. Copyright 2025, copyright Elsevier B.V.

**Figure 5:**
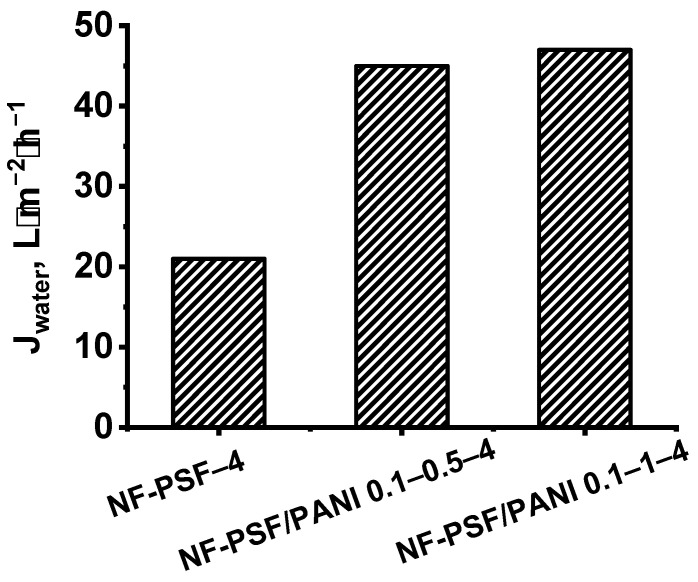
Dependence of water permeation (J_water_) of TFC NF membranes on the time of application of the PANI intermediate layer (PA layer—4 wt.% PIP, 4 wt.% CSA, 0.24 wt.% TMC) at ΔP = 0.5 MPa.

At the first stage, the influence of the duration of the deposition of the PANI interlayer from 0.1 wt.% ANI solution on the structure and performance of TFC NF membranes was studied (Figure 4 and Figure 5).

It was found that the application of the PANI interlayer on the surface of the PSF membrane substrate from 0.1 wt.% ANI solution for 0.5 h and 1 h resulted in a substantial decrease in the thickness of the PA layer from 104 nm down to 50 and 31 nm, respectively (Figure 4). When the PANI interlayer was applied for 0.5 h and 1 h from 0.1 wt.% ANI solution, the formation of smaller PA globules on the selective layer surface of TFC NF membranes compared to the reference TFC NF membrane occurred (Figure 4). It was found that the application of the PANI interlayer and the increase in the duration of the interlayer deposition led to a substantial decrease in the surface roughness parameters (Table 3). This is due to the hydrophilization of the membrane substrate selective layer when PANI was deposited, which led to its better impregnation by PIP solution. It is supposed that when the hydrophilic PANI layer is adsorbed on the pore walls, the capillary effect of the substrate is increased, and more PIP molecules are entrapped inside the pores. Moreover, the rate of PIP release from the PSF/PANI membrane substrate is decreased, yielding a lower rate of IP reaction. This leads to the formation of a PA layer with higher uniformity, lower surface roughness, and lower thickness in comparison with the initial TFC membrane (Figure 4, Table 3).

Moreover, it was shown that the application of the PANI interlayer led to the significant hydrophilization of the selective layer of TFC NF membranes due to the incorporation of highly hydrophilic PANI groups into the PA layer (Table 3). It was found that the water contact angle of the selective layer surface of TFC NF membranes decreased from 28 ± 2° down to θ < 10° when the PANI interlayer was deposited, regardless the time of deposition.

The nanofiltration performance of TFC membranes depending on the duration of the PANI layer application was studied using aqueous solutions of salts (Figure 5, Table 4). It was found that the formation of an intermediate layer based on PANI led to a significant increase in water permeation (J) of TFC NF membranes by more than two times: up to 45 and 47 L·m^−2^·h^−1^ (at ΔP = 0.5MPa) for NF-PSF/PANI 0.1–0.5–4 and NF-PSF/PANI 0.1–1–4 membranes, respectively, compared to 21 L·m^−2^·h^−1^ for the reference NF-PSF–4 membrane. This was due to the decrease in the selective layer thickness as well as substantial hydrophilization of the selective layer when the PANI interlayer was applied and the time of PANI deposition increased (Figure 4, Table 3). However, it was revealed that the permeability of the TFC NF membranes with a PANI interlayer did not change much with the rise in the duration of PANI layer application from 0.5 to 1 h (Figure 5).

It was shown that according to the rejection coefficients, salts can be arranged as follows for both the reference membrane and TFC membranes with a PANI interlayer:

MgSO_4_ > MgCl_2_ > CaCl_2_ > Na_2_SO_4_ > NaCl ≈ LiCl

It was found that the depositions of the PANI intermediate layer for 0.5 h resulted in a slight increase in the rejection coefficients of divalent salts (MgSO_4_, MgCl_2_, and CaCl_2_) compared to the reference NF-PSF–4 membrane without intermediate layer (Table 4). However, the selectivity of monovalent salts NaCl and LiCl was shown to decrease, and selectivity of Na_2_SO_4_ was not changed compared to the reference membrane (Table 4). The reason for the increased selectivity may be the formation of a more uniform and more crosslinked selective layer when the PANI interlayer was applied for 0.5 h. Thus, when the PANI interlayer was deposited for 0.5 h, both the permeation and selectivity of the TFC membrane increased compared to the reference membrane.

With an increase in PANI interlayer application time from 0.5 h to 1 h, a looser PA layer was probably formed that caused a reduction in the rejection coefficient (R) for MgSO_4_ from 99.99% to 93%, for MgCl_2_ from 99 to 92%, for CaCl_2_ from 92 to 91%, and for NaCl and LiCl from 25% to 22% for membranes with PANI interlayers applied for 0.5 and 1 h, respectively (Table 4). However, MgSO_4_ rejection of the NF-PSF/PANI 0.1–1–4 TFC membrane is slightly higher compared to the reference TFC membrane (Table 4). It is worth noting that rejection of the NF-PSF/PANI 0.1–1–4 membrane is practically at the same level as for the reference membrane; however, the water permeation for NF-PSF/PANI 0.1–1–4 is significantly higher compared to the reference membranes.

It was found that SDM rejection slightly increases when the PANI interlayer was deposited for 0.5 h compared to the reference TFC NF membrane. Application of the PANI interlayer for 1 h led to a substantial decrease in SDM rejection compared to both NF-PSF-4 and NF-PSF/PANI 0.1–0.5–4 membranes.

According to the obtained results, 0.5 h was selected as the optimal duration for PANI layer application for further experiments.

#### 3.2.2. The Effect of the Concentration of ANI, PIP, and TMC on the Structure and Properties of TFC NF Membranes

**Figure 6:**
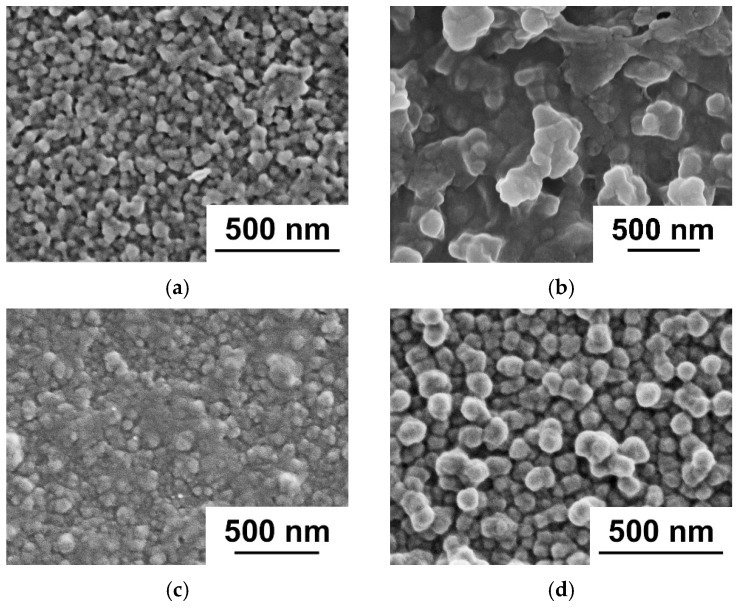

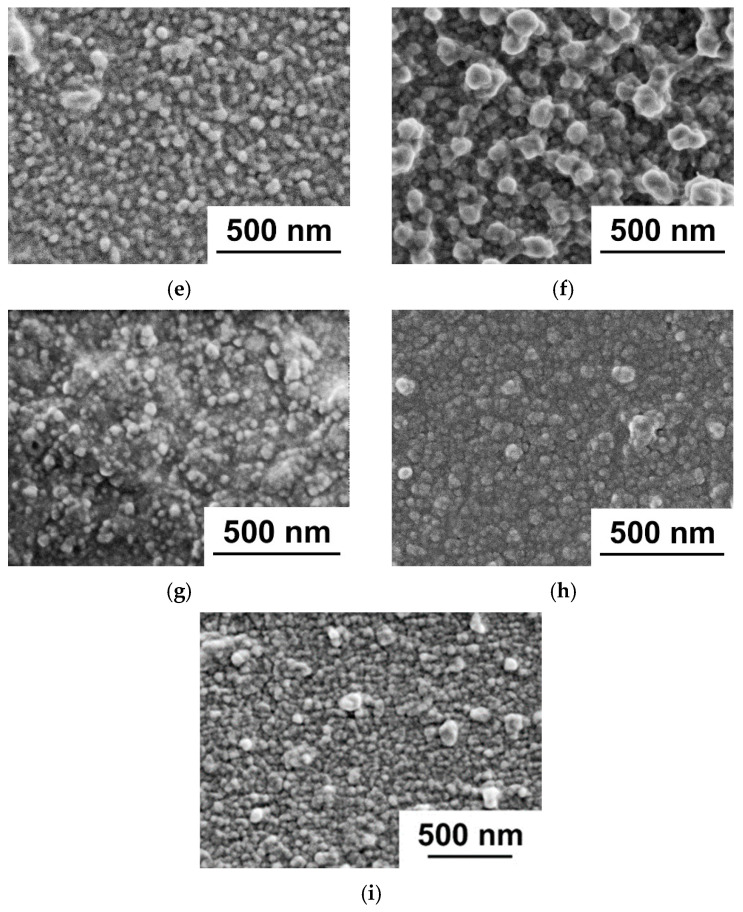
SEM microphotographs of the selective layer surface of TFC NF membranes with different ANI, PIP, and TMC concentrations upon PANI interlayer and PA selective layer formation: (**a**) NF-PSF–2; (**b**) NF-PSF–4; (**c**) NF-PSF/PANI 0.05–0.5–2; (**d**) NF-PSF/PANI 0.05–0.5–4; (**e**) NF-PSF/PANI 0.1–0.5–2; (**f**) NF-PSF/PANI 0.1–0.5–4; (**g**) NF-PSF/PANI 0.3–0.5–2; (**h**) NF-PSF/PANI 0.3–0.5–4; (**i**) NF-PSF/PANI 0.1–0.5–1.

**Figure 7:**
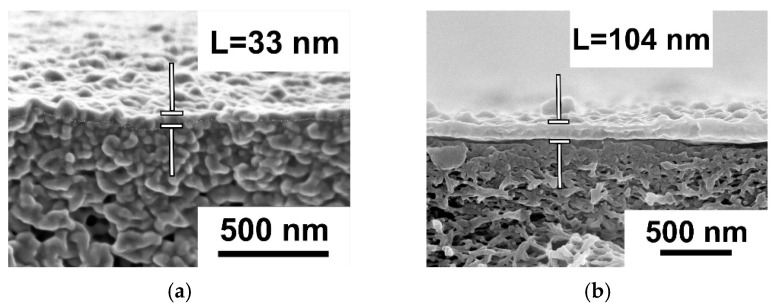

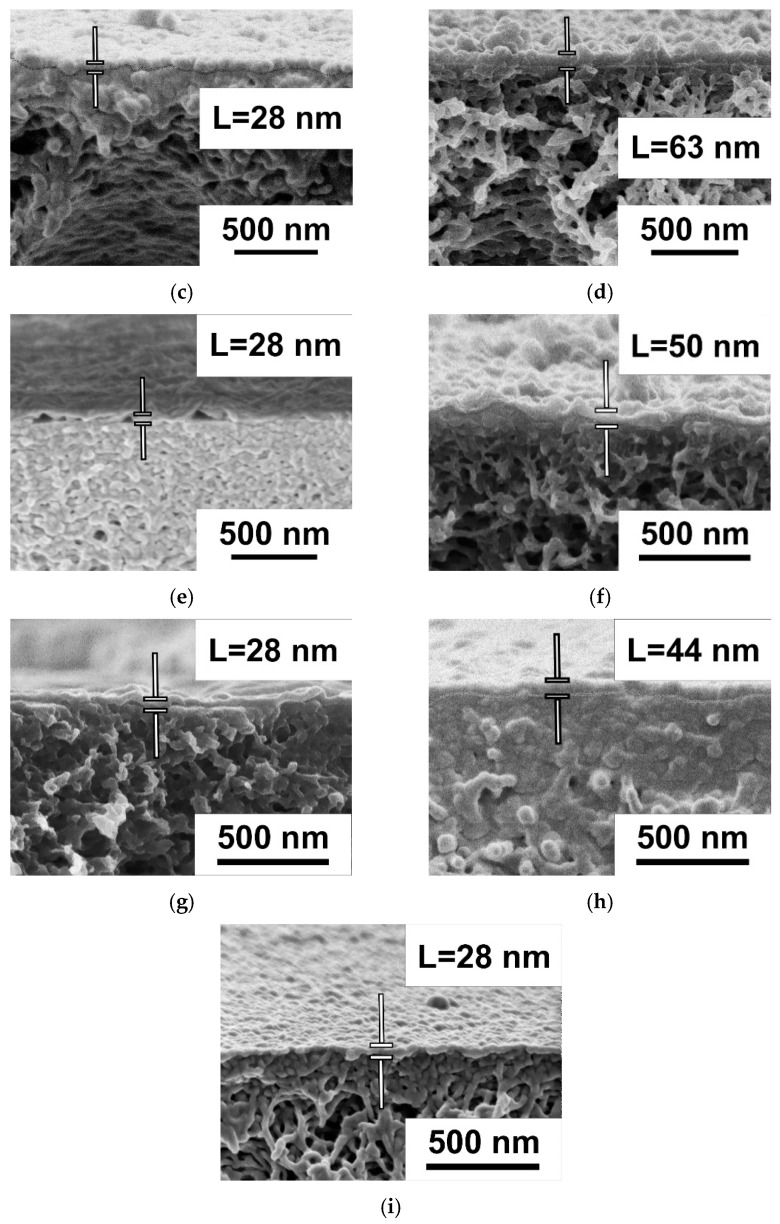
SEM microphotographs of the cross-sections of TFC NF membranes depending on the ANI concentration under different compositions of the PA selective layer: (**a**) NF-PSF–2; (**b**) NF-PSF–4; (**c**) NF-PSF/PANI 0.05–0.5–2; (**d**) NF-PSF/PANI 0.05–0.5–4; (**e**) NF-PSF/PANI 0.1–0.5–2; (**f**) NF-PSF/PANI 0.1–0.5–4; (**g**) NF-PSF/PANI 0.3–0.5–2; (**h**) NF-PSF/PANI 0.3–0.5–4; (**i**) NF-PSF/PANI 0.1–0.5–1.

**Figure 8:**
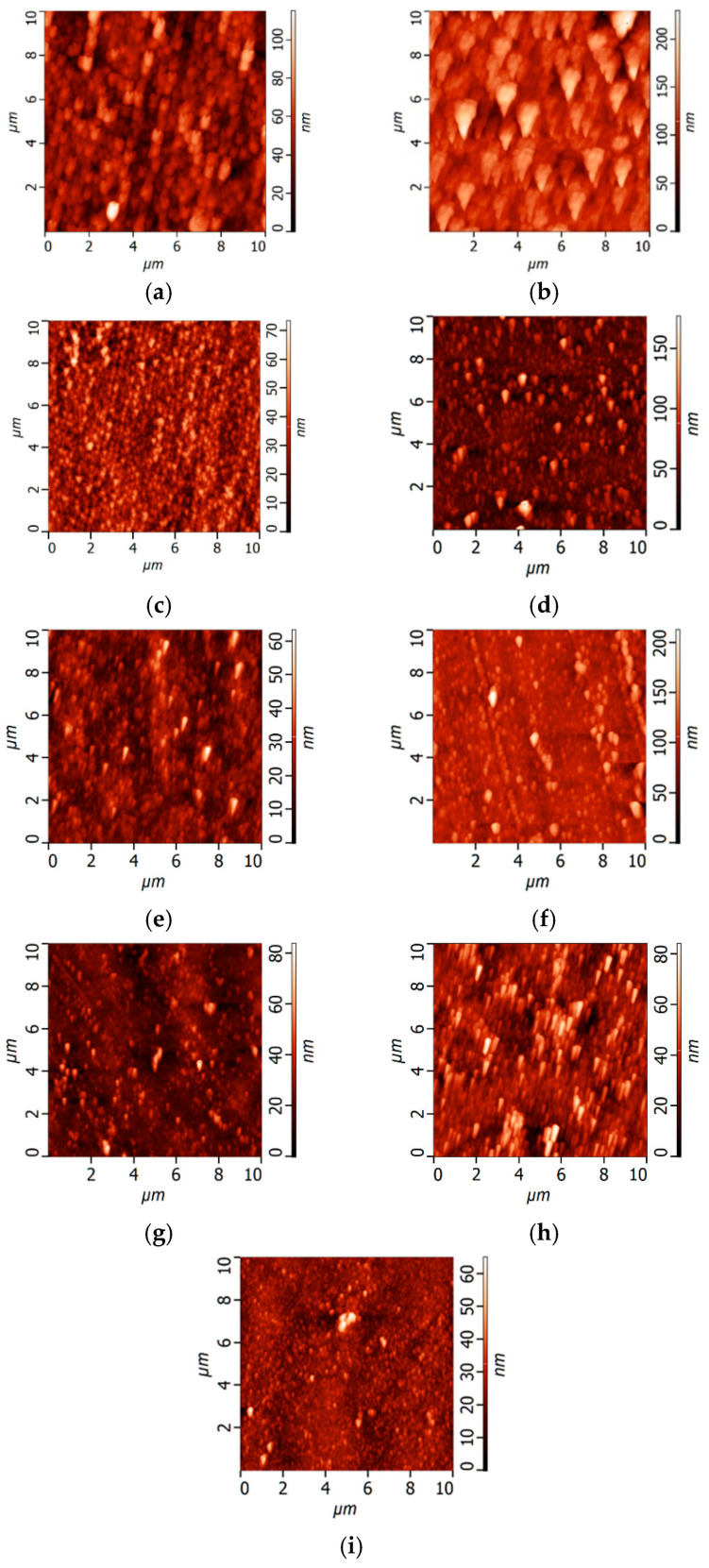
AFM microphotographs of the selective layer surface NF/PANI 0.5 h/PA membranes depending on the ANI concentration under different compositions of the PA selective layer: (**a**) NF-PSF–2; (**b**) NF-PSF–4; (**c**) NF-PSF/PANI 0.05–0.5–2; (**d**) NF-PSF/PANI 0.05–0.5–4; (**e**) NF-PSF/PANI 0.1–0.5–2; (**f**) NF-PSF/PANI 0.1–0.5–4; (**g**) NF-PSF/PANI 0.3–0.5–2; (**h**) NF-PSF/PANI 0.3–0.5–4; (**i**) NF-PSF/PANI 0.1–0.5–1.

**Figure 9:**
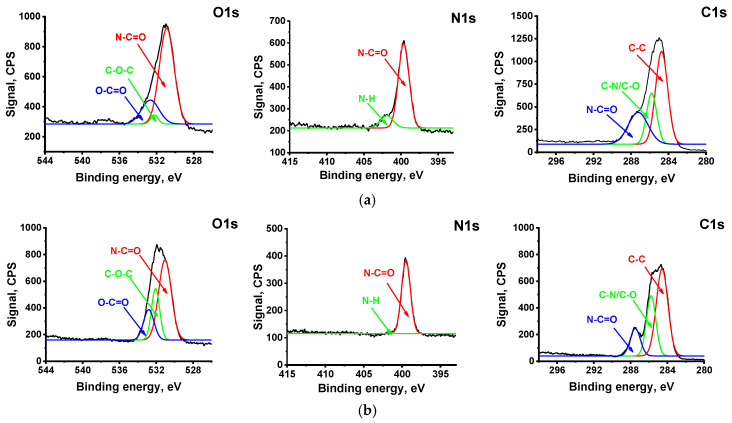
The high-resolution XPS spectra of the selective layer surface of TFC NF membranes: (**a**) NF-PSF–4; (**b**) NF-PSF/PANI 0.1–0.5–4.

The effect of the ANI concentration (0.05 wt.%, 0.1 wt.%, 0.3 wt.%, and 0.5 wt.%) in the aqueous solution applied to form the intermediate layer on the performance of TFC NF membranes was studied. The duration of PANI interlayer deposition was 0.5 h. The effect of the concentration of monomers in IP reaction during the formation of the selective PA layer on the structure and performance of TFC NF membranes with a PANI interlayer obtained by oxidative polymerization was investigated (Figure 6, Figure 7, Figure 8 and Figure 9, Table 5 and Table 6). The PA layer was formed using three different concentrations of PIP, namely 1%, 2%, and 4%, and three different concentrations of TMC, namely 0.06%, 0.12%, and 0.24%, respectively.

SEM microphotographs of the selective layer surface and cross-sections of TFC NF membranes with different ANI, PIP, and TMC concentrations used for PANI interlayer and PA selective layer formation are shown in Figure 6 and Figure 7. AFM images and surface roughness parameters of the developed TFC NF membranes are presented in Figure 8 and Table 5.

As discussed above, when the ANI concentration in oxidative polymerization increased, the surface roughness and hydrophilicity of the selective layer surface of the membrane substrate were enhanced and the pore size declined (Section 3.1, Figure 1 and Figure 2, Table 2).

It was found that the surface of the selective layer of all studied TFC membranes is characterized by the presence of PA globular formations, which are the typical structure of TFC NF membranes based on piperazine amide (Figure 6). Application of a PANI interlayer and an increase in ANI concentration in oxidative polymerization lead to a significant decrease in the size of PA globules. Moreover, the shape of PA globules becomes more regular and the size distribution narrows when the ANI concentration in oxidative polymerization increases both for membranes prepared using 2% PIP and 4% PIP (Figure 6). It was revealed that an increase in the concentrations of PIP and TMC yields an increase in the size of PA globules on the selective layer surface due to the increase in the number of molecules that participate in the IP reaction (Figure 6e,f,i).

It was shown that the application of a PANI interlayer yields a decrease in the thickness of the PA selective layer both for TFC membranes prepared using 2% PIP and 4% PIP (Figure 7). It was found that an increase in ANI concentration from 0.05 wt.% up to 0.3 wt.% during PANI interlayer formation results in a decrease in the thickness of the PA selective layer for TFC NF membranes prepared using 4 wt.% PIP from 63 down to 44 nm (Figure 7b,d,h,f). However, no changes in PA layer thickness occurred for TFC NF membranes prepared using 2 wt.% PIP when the ANI concentration rose (Figure 7c,e,g). As expected, an increase in monomer concentrations in IP leads to an increase in the PA selective layer thickness regardless of ANI concentration and the presence or absence of a PANI interlayer (Figure 7). For instance, the thickness of the PA selective layer was shown to increase slightly from 28 up to 50 nm with the rise in PIP concentration from 1 to 4 wt.% and TMC concentration from 0.06 wt.% up to 0.24 wt.% correspondingly (Figure 7e,f,i).

It was found that surface roughness parameters of TFC NF membranes significantly declined with the increase in the concentration of ANI during oxidative polymerization applied for PANI interlayer formation (Figure 8, Table 5).

As expected, surface roughness parameters increased when PIP and TMC concentrations used for PA layer formation increased for all studied TFC membranes (Figure 8, Table 5). This is due to the increase in the size of PA globules when the PIP concentration increases from 1 wt.% up to 4 wt.%, which is clearly observed by SEM (Figure 6e,f,i). Thus, a thicker and rougher selective layer was formed with the increase in PIP and TMC concentrations, which is in good agreement with the literature data [63,64].

Overall, with the increase in PIP and TMC concentrations, interface instability increases due to intensified IP reaction, causing a concurrent increase in the thickness and roughness of the PA selective layer. It was reported that the crosslinking degree of the PA selective layer also depends on amine and acyl chloride concentrations during IP. It was shown that the degree of crosslinking of the PA selective layer increased from 78.6% to 98.8% when the concentration of m-phenylenediamine increased from 0.1 wt.% to 10 wt.%. This is due to the sufficient supply of monomers at high concentration, which promoted the migration of amine from the aqueous phase to the organic phase. As a result, more corrugated and thicker PA selective layers were formed via IP [64]. It was found that independent of the monomer concentrations, a highly hydrophilic PA selective layer was formed when the PANI interlayer was applied, which is due to the incorporation of hydrophilic groups of PANI in the selective layer (Table 5).

The changes in TFC membrane structure when the ANI concentration used for PANI interlayer formation increases are attributed to the enhancement of hydrophilicity and decline in the pore size of the membrane substrate selective layer (Figure 1 and Figure 2, Table 2). Moreover, the formation of hydrogen bonds and Van der Waals bonds between PIP and PANI facilitates the storage of more PIP molecules in the pores of the membrane substrate. However, the rate of PIP release to the IP reaction interface decreases, which leads to the formation of a more uniform, thinner, and smoother PA selective layer when the ANI concentration increases during PANI interlayer formation. On the other hand, the constant thickness (28 nm) of the PA selective layer regardless of ANI concentration when a lower concentration (2 wt.%) of PIP is used may be due to the insufficient number of PIP molecules at the IP reaction aqueous–organic interface attributed to their retarded release from the PANI interlayer.

The compositions of the PA selective layer of the reference NF-PSF–4 membrane and NF-PSF/PANI 0.1–0.5–4 TFC membrane with a PANI interlayer were studied using XPS analysis (Figure 9, Table 6).

Figure 9 shows the high-resolution XPS spectra C1s, O1s, and N1s of the selective layer surface of the initial TFC membrane and TFC membranes with a PANI interlayer.

The O1s spectrum was resolved into three typical peaks at 533.2, 532.2, and 530.9 eV, which were attributed to O–C=O, C–O–C, and N–C=O groups, respectively (Figure 9). It is generally accepted that O=C–O groups are generated due to the hydrolysis of unreacted acyl chloride groups. It should be noted that the C–O–C peak can be assigned to the ether groups of PSF, which is a main membrane substrate material.

It was demonstrated that the N1s spectrum can be deconvoluted into two characteristic peaks at around 399.5 eV and 400.9 eV, which were attributed to N–C=O and N–H groups, respectively (Figure 9).

The C1s spectra have N–C=O, C–N/C–O, and C–H/C–C groups at 287.6, 286.0, and 284.8 eV, respectively (Figure 9).

The clear peaks for C, O, and N elements occurred in XPS spectra (Figure 9). There are two sources of N in the XPS spectra: PA and PANI. It was found that the concentration of N and O atoms rose when a PANI interlayer was applied (Table 7). The overall nitrogen concentration enhanced since PANI macromolecules were incorporated into the PA layer, which is indirectly proved by the significant rise in the PA layer’s hydrophilicity (Table 5 and Table 6). It was found that the total content of C atoms decreased since the concentration of N and O atoms rose (Table 6).

The ratios of the peak areas N–C=O/O–C=O (in O 1s spectra) and N–C=O/NH (in N 1s spectra) were calculated. It was demonstrated that the amount of the amide groups rose when the PANI interlayer was deposited (Table 6), which indicated that there was a smaller amount of free unreacted carboxylic and amine groups in the PA layer. This confirms that the formation of the PANI interlayer leads to the formation of a PA layer with a higher crosslinking degree (Table 6).

**Figure 10:**
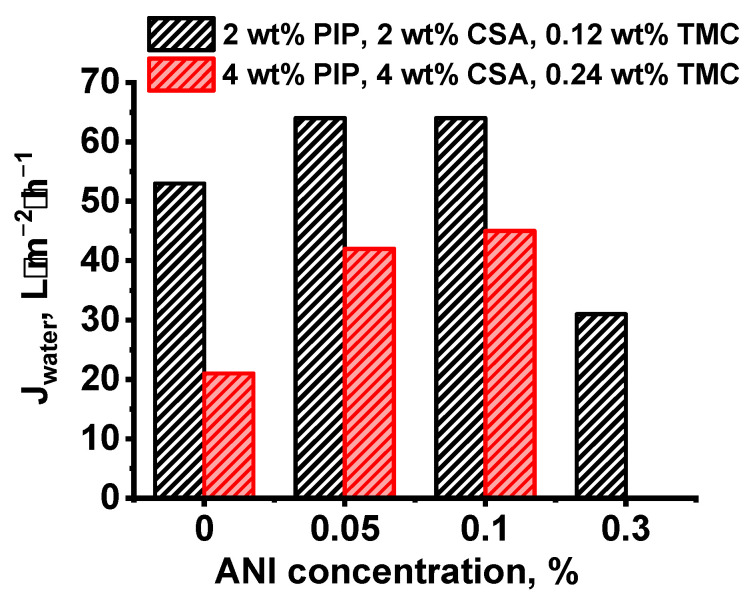
Water permeation (at ΔP = 0.5 MPa) of TFC NF membranes versus the concentration of ANI in aqueous solution.

It was shown that water permeation substantially increased when 0.05 and 0.1 wt.% ANI in the aqueous solution was applied for PANI interlayer deposition both for 2 wt.% and 4 wt.% PIP solutions used for PA layer formation compared to the reference NF-PSF–2 and NF-PSF–4 membranes. The best water permeation was achieved for NF-PSF/PANI 0.05–0.5–2 and NF-PSF/PANI 0.1–0.5–2 membranes and reached 64 L·m^−2^·h^−1^ at ΔP = 0.5 MPa (Figure 10). The rise in water permeation with the increase in ANI concentration up to 0.05 and 0.1 wt.% is due to the decrease in PA selective layer thickness, which was clearly observed by SEM (Figure 7). The TFC NF membrane series with 2 wt.% PIP demonstrated higher water permeation compared to the TFC NF membrane series with 4 wt.% PIP due to the smaller selective layer thickness (Figure 10). It was revealed that an increase in the concentration of ANI up to 0.3 wt.% yielded a decline in water permeability down to 31 L·m^−2^·h^−1^ for the NF-PSF/PANI 0.3–0.5–2 membrane in spite of the thin PA layer (28 nm) (Figure 7 and Figure 10). Moreover, it was found that the formation of the PANI intermediate layer from a solution with a monomer concentration of 0.5 wt.% with subsequent application of a PA layer resulted in the preparation of an impermeable TFC NF membrane at ΔP = 0.5 MPa. However, when 0.3 wt.% ANI was used for PANI interlayer formation, NF-PSF/PANI 0.3–0.5–4 was also impermeable. This may be due to the increased mass transfer resistance due to the blockage of pores of the selective layer of the membrane substrate and formation of a thick PANI interlayer, which counterbalances the thin PA layer. This is proved by the substantial decrease in the pure water flux of the membrane substrate UF-PSF/PANI 0.3 down to 5 L·m^−2^·h^−1^ at ΔP = 0.1 MPa compared to 180 L·m^−2^·h^−1^ for the reference membrane (Figure 3).

Thus, the increase in ANI concentration during PANI interlayer formation up to 0.05 and 0.1 wt.% leads to a rise in water permeation due to the increase in PA layer thickness. However, a further increase in ANI concentration up to 0.3 and 0.5 wt.% decreases water permeation, which may be attributed to the blockage of the pores of the membrane substrate, which increases mass transfer resistance.

It was found that an increase in the concentration of monomers in the IP led to a decrease in water permeation from 64 L·m^−2^·h^−1^ for the NF-PSF/PANI 0.1–0.5–1 and NF-PSF/PANI 0.1–0.5–2 membranes down to 45 L·m^−2^·h^−1^ for the NF-PSF/PANI 0.1–0.5–4 membrane (at ΔP = 0.5 MPa) (Figure S3). The decrease in water permeation was due to the formation of a thicker and denser selective PA layer (Figure 7e,f,i). However, the effect of the increased selective layer thickness is counterbalanced by the significant increase in surface roughness parameters when the concentration of PIP and TMC rises in the selective layer (Table 5).

It was found that rejection of MgSO_4_ significantly increases up to 99.99% when 0.05 and 0.1 wt.% ANI is applied for PANI interlayer formation both for 2 wt.% PIP and 4 wt.% PIP TFC NF membrane series (Table 8).

However, the rejection of MgSO_4_ for NF-PSF/PANI 0.3–0.5–2 was found to be 71% compared to the 82% for the reference NF-PSF–2 membrane. So, an increase in ANI concentration up to 0.3 wt.% yields a decrease in MgSO_4_ rejection for the TFC NF membrane series with 2 wt.% PIP. Na_2_SO_4_ and MgCl_2_ rejection increased with the maximum for NF-PSF/PANI 0.05–0.5–2 and NF-PSF/PANI 0.1–0.5–4 TFC NF membranes (at 0.05 wt.% ANI for membranes with 2 wt.% PIP and 0.1 wt.% for membranes with 4 wt.% PIP). The rejection coefficients for CaCl_2_ and SDM decreased with increasing ANI concentration for all developed TFC NF membranes with 2 wt.% PIP used for PA layer formation (Table 8). Rejection coefficients for monovalent salts NaCl and LiCl were revealed to slightly decrease for the NF-PSF/PANI 0.05–0.5–2 membrane and increase for the NF-PSF/PANI 0.1–0.5–2 membrane and NF-PSF/PANI 0.3–0.5–2 membrane compared to the reference NF-PSF–2 membrane (Table 8). Thus, the better rejection demonstrates the NF-PSF/PANI 0.05–0.5–2 membrane compared to the NF-PSF–2 membrane.

It was shown that the rejection coefficient for MgSO_4_ did not change with the variation in the concentrations of PIP and TMC during PA layer formation via IP and was 99.99% (compare the rejection of NF-PSF/PANI 0.1–0.5–1, NF-PSF/PANI 0.1–0.5–2, and NF-PSF/PANI 0.1–0.5–4 membranes, Table 8). Overall, the rise in the concentration of monomers in the IP reaction led to a significant increase in rejections for all studied salts and antibiotic SDM.

According to the rejections for NF-PSF/PANI 0.1–0.5–1 and NF-PSF/PANI 0.1–0.5–2, salts can be arranged as follows:

MgSO_4_ > MgCl_2_ > Na_2_SO_4_ > CaCl_2_ > NaCl > LiCl

However, with the increase in PIP and TMC concentration, this line slightly changes for the NF-PSF/PANI 0.1–0.5–4 membrane:

MgSO_4_ > MgCl_2_ > CaCl_2_ > Na_2_SO_4_ > NaCl > LiCl

When 4 wt.% PIP is used for PA layer formation, an increase in ANI concentration results in an increase in rejection of all studied salts with the maximum values at 0.1 ANI concentration. This is due to the formation of a PA layer with a higher crosslinking degree (with a higher number of amide groups) when the PANI intermediate layer (0.3 wt.% ANI concentration) was deposited, which was confirmed by XPS (Figure 9, Table 6).

The pattern of change in rejection with the increase in PIP and TMC concentration suggests that at lower monomer concentrations, a PA selective layer with a lower crosslinking degree is formed. This may be attributed to the insufficient supply of monomers at lower concentrations (1 wt.% PIP and 2 wt. % PIP) to the interface of the aqueous and organic phases in the IP reaction. Moreover, as discussed above, a hydrophilic PANI interlayer retards the release of PIP to the reaction interface, leading to an insufficient number of amine molecules in the reaction zone.

The zeta potential of the selective layer surface of NF membranes was determined using an electrokinetic analyzer, and the results are presented in Table 7. It was found that the formation of a PANI interlayer on the surface of PSF membranes via oxidation polymerization yielded an increase in the positive zeta potential at pH 3 and negative zeta potential at pH 7 and 10 (Table 7). Moreover, it was shown that the NF-PSF–2 membrane is characterized by a higher positive zeta potential value at pH 3 compared to the NF-PSF–4 membrane. It was revealed that application of a PANI interlayer yields a slight rise in the isoelectric point of the membrane surface from 4.0 for the reference NF-PSF–4 membrane up to 4.4 for the NF-PSF/PANI 0.1–0.5–4 membrane and from 4.4 for the NF-PSF–2 membrane up to 4.5 for the NF-PSF/PANI 0.05–0.5–2 membrane. The increase in positive zeta potential values at pH 3 and the rise in isoelectric point are due to the protonation of PANI in acidic media, since PANI groups are incorporated in the PA selective layer. The increase in negative zeta potential values at pH 7 and 10 can be attributed to the increase in binding sides for non-specific adsorption of hydroxide and chloride ions during zeta potential measurements.

The results of the investigation of Mg^2+^/Li^+^ separation are presented in Table 9. It was found that the formation of a PANI interlayer yielded a significant enhancement of the Mg^2+^/Li^+^ separation factor by more than four and seven times for the NF-PSF/PANI 0.05–0.5–2 and NF-PSF/PANI 0.1–0.5–4 membranes, respectively, compared to the reference NF-PSF–2 and NF-PSF–4 membranes.

**Figure 11:**
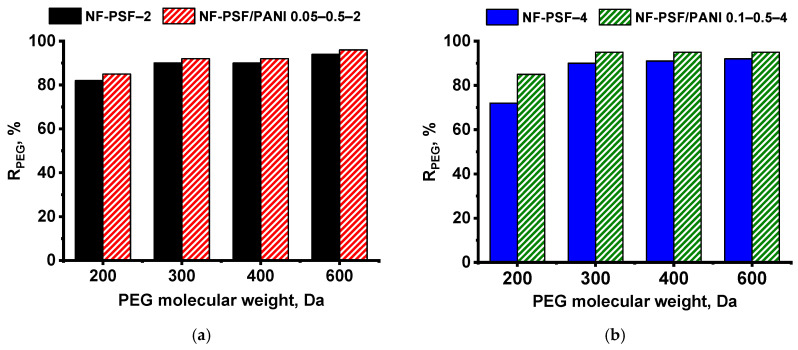
Rejection coefficients of PEGs of different molecular weights of the developed TFC NF membranes. PIP aqueous solution concentration, wt.%: (**a**) 2, (**b**) 4.

The rejection coefficients of PEGs of the developed TFC NF membranes are presented in Figure 11 It was shown that rejection coefficients for PEGs of different molecular weights were higher for PANI-modified TFC NF membranes in both cases, prepared using 2 wt.% PIP and 4 wt.% PIP aqueous solutions (Figure 11). The rejection coefficients of TFC NF membranes for PEG 200 Da were found to be 72–85 wt.%. The rejection coefficients of PEG 300, 400, and 600 Da were over 90% (Figure 11).

**Figure 12:**
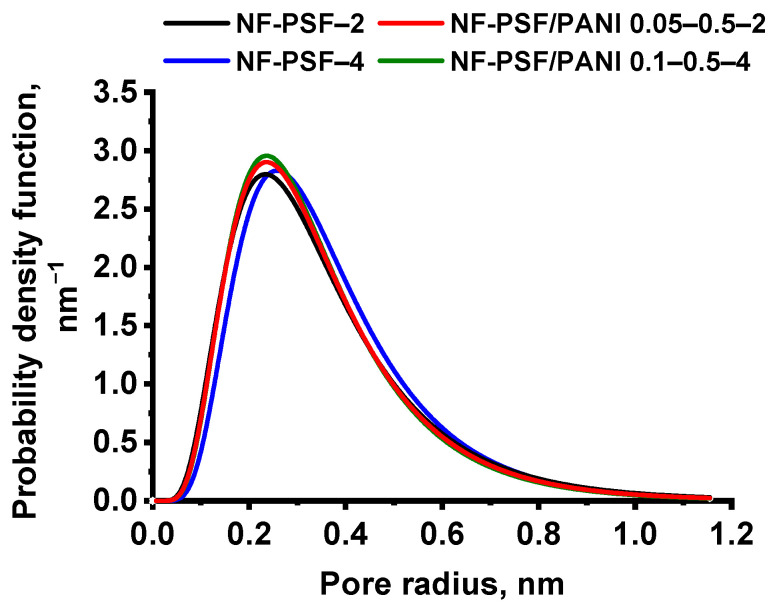
Pore size distribution of TFC NF membranes.

According to the obtained results, the MWCO and average pore size as well as pore size distribution of TFC NF membranes were measured and calculated (Table 10, Figure 12). It was revealed that application of a PANI interlayer caused a decrease in the average pore size together with a decline in the MWCO of the developed membranes compared to the corresponding reference NF-PSF–2 and NF-PSF–4 TFC membranes (Table 10).

The mean effective pore size of the obtained TFC NF membranes was evaluated using PEG 200–600. The pore size distribution of NF membranes is shown in Figure 12. It was found that the formation of a PANI interlayer led to a slight narrowing the pore size distribution compared to the reference NF-PSF–2 and NF-PSF–4 membranes (Figure 12). This can be explained by better impregnation of the PSF/PANI membrane substrate with PIP, caused by the increase in its hydrophilicity, and as a result the formation of a PA selective layer with a higher crosslinking degree and smaller pores (Figure 12, Table 6 and Table 10).

The results on average pore size and pore size distribution studies are in good accordance with the MWCO and rejection coefficients studies (Table 7, Table 9 and Table 10, Figure 11 and Figure 12). The increased selectivity of the developed NF-PSF/PANI 0.05–0.5–2 and NF-PSF/PANI 0.1–0.5–4 membranes (especially for MgCl_2_) is due to the decrease in pore size and narrowing of the pore size distribution of the selective layer. Moreover, the increase in the negative zeta potential at pH 7 and 10 decreases the transport of chloride ions of MgCl_2_ providing higher MgCl_2_ rejection_._ The transport of chloride ions may decrease the rejection of MgCl_2_ since Mg^2+^ may be transported through the membrane to provide the electroneutrality of the solution. It often leads to lower MgCl_2_ rejection compared to MgSO_4_ and Na_2_SO_4_ (Table 7).

**Figure 13:**
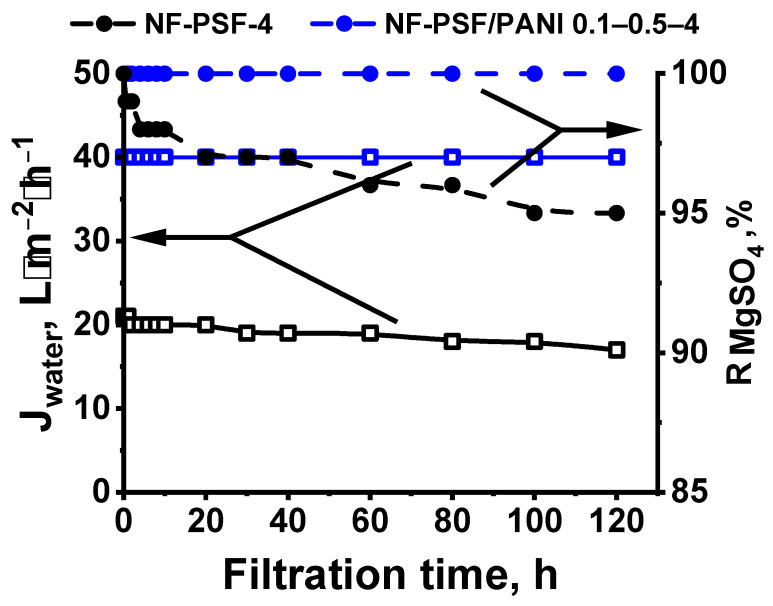
Long-term performance of NF-PSF–4 and NF-PSF/PANI 0.1–0.5–4 membranes (ΔP = 0.5 MPa).

According to the optimal combination of water permeation and selectivity, NF-PSF/PANI 0.1–0.5–4 was selected for further studies of long-term operation performance. The long-term operation performance of the NF-PSF–4 and NF-PSF/PANI 0.1–0.5–4 membrane during the filtration of water and aqueous solution of magnesium sulphate (2 g·L^−1^) at ΔP = 0.5 MPa is presented in Figure 13. It was revealed that the water permeance of the TFC NF membrane as well as the MgSO_4_ rejection coefficient were constant for 120 h. Based on the obtained data, it could be concluded that the TFC NF membrane modified with application of a PANI intermediate layer is characterized by high stability during 120 h of the nanofiltration experiment.

The effect of chemical treatment on the performance of the developed membrane after oxidation with sodium hypochlorite solution and acid treatment was investigated (Table 11). It was found that after chemical treatment, the water permeation at ΔP = 0.5MPa slightly increased for the reference NF-PSF–2 and NF-PSF–4 membranes but remained unchanged for TFC NF membranes with a PANI interlayer (Table 11). Moreover, the rejection coefficients for MgSO_4_ were also the same (>99.99%) for modified TFC NF-PSF/PANI 0.05–0.5–2 and NF-PSF/PANI 0.1–0.5–4 membranes but slightly declined for reference NF-PSF–2 and NF-PSF–4 membranes (Table 11). Thus, it was found that the developed TFC NF membranes possessed better chemical resistance compared to the reference membranes.

The nanofiltration performance of the developed TFC NF membranes was compared to the transport characteristic of membranes reported so far (Table 12). It was demonstrated that the pure water permeance, MgSO_4_ and Na_2_SO_4_ rejection, and Mg^2+^/Li^+^ separation factor of PSF/PANI/PA nanofiltration membranes surpass most of TFC NF membranes with an interlayer reported previously.

## 4. Conclusions

The principles of PA selective layer formation via interfacial polymerization on PSF ultrafiltration membrane substrates with a PANI interlayer were revealed. It was discovered that the conditions of PANI interlayer application—such as the duration of PANI layer formation and the concentration of aniline—significantly affect the structure and performance of the resulting TFC nanofiltration membranes. It was found that deposition of a PANI layer via oxidative polymerization on the surface of a PSF ultrafiltration membrane yields partial pore blocking, an increase in surface roughness, and hydrophilization of the membrane selective layer. When a higher aniline concentration and longer time of PANI layer formation are applied, a rougher and more hydrophilic selective layer of the membrane substrate with smaller pores is formed. These substantial changes of structure, porosity, and hydrophilic–hydrophobic properties of the membrane selective layer affected the PA layer formation via IP during TFC membrane preparation. It was supposed that the hydrophilic PANI layer covering the pore walls of the membrane substrate enhances the capillary effect of the substrate, and more amine monomers can be absorbed within the pores. Moreover, the release of PIP from the highly hydrophilic membrane substrate coated with a PANI layer is retarded, leading to a lower rate of IP reaction, which results in the formation of more uniform, thinner, and smoother PA layer compared to the reference TFC membrane.

The developed method improves TFC membrane water permeation up to 45–64 L·m^−2^·h^−1^ at 0.5 MPa and increases selectivity, with rejection coefficients for MgSO_4_ exceeding 99.99%, for LiCl ranging from 5 to 25%, and for sodium sulfadimetoxine ranging from 80 to 95%. Moreover, the Mg^2+^/Li^+^ separation factor of the developed TFC NF membranes with a PANI interlayer was found to be 37 and 58 compared to 8 and 9 for reference membranes. Additionally, this approach ensures improved long-term operational stability of TFC nanofiltration membranes with a PANI interlayer.

## Figures and Tables

**Table 1:** The codes of UF and NF membranes.

Membrane Code	PANI Interlayer		PA Selective Layer	
	ANI Concentration, wt.%	Duration of PANI Layer Application, h	PIP Concentration, wt.%	TMC Concentration, wt.%
UF-PSF	-			
UF-PSF/PANI 0.05	0.05	0.5	-	
UF-PSF/PANI 0.1	0.1		-	
UF-PSF/PANI 0.3	0.3		-	
UF-PSF/PANI 0.1–1	0.1	1.0	-	
NF-PSF–2	-		2.0	0.12
NF-PSF–4			4.0	0.24
NF-PSF/PANI 0.05–0.5–2	0.5	0.5	2.0	0.12
NF-PSF/PANI 0.05–0.5–4			4.0	0.24
NF-PSF/PANI 0.1–0.5–1	0.1		1.0	0.06
NF-PSF/PANI 0.1–0.5–2			2.0	0.12
NF-PSF/PANI 0.1–0.5–4			4.0	0.24
NF-PSF/PANI 0.1–1–4		1.0		
NF-PSF/PANI 0.3–0.5–2	0.3	0.5	2.0	0.12
NF-PSF/PANI 0.3–0.5–4			4.0	0.24
NF-PSF/PANI 0.5–0.5–4	0.5			

**Table 2:** The surface roughness parameters and water contact angles of PSF UF membranes with a PANI layer obtained by physical adsorption in the process of oxidative polymerization of ANI for 0.5 h and 1 h.

Membrane Code	Roughness Parameters		Water Contact Angle, °
	R_a_, nm	R_q_, nm	
UF-PSF	1.87	2.32	65 ± 2
UF-PSF/PANI 0.05	2.25	2.95	49 ± 2
UF-PSF/PANI 0.1	2.19	2.74	38 ± 2
UF-PSF/PANI 0.3	5.46	7.88	26 ± 2
UF-PSF/PANI 0.1–1	3.94	5.24	30 ± 2

**Table 3:** The surface roughness parameters of TFC NF membranes with a PANI interlayer obtained from 0.1 wt.% ANI solution.

Membrane Code	Roughness Parameters		Water Contact Angle, °
	R_a_, nm	R_q_, nm	
NF-PSF–4	18.95	25.10	28 ± 2
NF-PSF/PANI 0.1–0.5–4	10.98	15.48	<10
NF-PSF/PANI 0.1–1–4	5.54	8.20	<10

**Table 4:** Salt and SDM rejection coefficients of TFC NF membranes with different times of application of the PANI intermediate layer (PA layer—4 wt.% PIP, 4 wt.% CSA, 0.24 wt.% TMC) at ΔP = 0.5 MPa.

Salt	Rejection		
	NF-PSF–4	NF-PSF/PANI 0.1–0.5–4	NF-PSF/PANI 0.1–1–4
MgSO_4_	92	99.99	93
Na_2_SO_4_	90	90	90
MgCl_2_	91	99	92
CaCl_2_	91	92	91
NaCl	28	25	22
LiCl	28	25	22
SDM	95	96	83

**Table 5:** The surface roughness parameters of TFC NF membranes with a PANI interlayer obtained for 0.5 h and PA layer.

Membrane Code	Roughness Parameters		Water Contact Angle, °
	Ra, nm	Rq, nm	
NF-PSF–2	10.51	13.54	28 ± 2
NF-PSF–4	18.95	25.10	30 ± 2
NF-PSF/PANI 0.05–0.5–2	6.87	8.58	<10
NF-PSF/PANI 0.05–0.5–4	12.35	17.56	<10
NF-PSF/PANI 0.1–0.5–2	6.53	9.07	<10
NF-PSF/PANI 0.1–0.5–4	10.98	15.48	<10
NF-PSF/PANI 0.3–0.5–2	5.68	7.85	<10
NF-PSF/PANI 0.3–0.5–4	8.39	11.05	<10
NF-PSF/PANI 0.1–0.5–1	4.07	5.60	<10

**Table 6:** Composition of PA selective layer and crosslinking degree of the developed TFC NF membranes studied by XPS.

Membrane	Atom Content in the Selective Layer of TFC NF Membranes, %			Ratio of Peak Intensities		Crosslinking Degree, %
	O	N	C	N–C=O/ O–C=O	N–C=O/ N–H	
NF-PSF–4	19	12	69	3.67	5.82	31.33
NF-PSF/PANI 0.1–0.5–4	20	14	66	3.93	97.9	47.06

**Table 7:** Zeta potential of the selective layer surface of TFC NF membranes.

Membrane Code	Zeta-Potential of Membrane Surface, mV			Isoelectric Point, pH
	pH 3	pH 7	pH 10	
NF-PSF–2	8.71	−32.55	−34.68	4.4
NF-PSF/PANI 0.05–0.5–2	15.06	−45.38	−52.22	4.5
NF-PSF–4	5.68	−32.49	−34.32	4.0
NF-PSF/PANI 0.1–0.5–4	14.94	−40.53	−46.67	4.4

**Table 8:** Rejection coefficients of NF membranes with different PA layer compositions.

Membrane Code	Rejection, %						
	MgSO_4_	Na_2_SO_4_	MgCl_2_	CaCl_2_	NaCl	LiCl	SDM
NF-PSF–2	82	70	90	86	16	6	83
NF-PSF/PANI 0.05–0.5–2	>99.99	84	96	79	10	5	80
NF-PSF/PANI 0.1–0.5–2	>99.99	76	84	59	24	10	78
NF-PSF/PANI 0.3–0.5–2	71	54	73	74	22	22	80
NF-PSF–4	92	90	91	91	28	28	95
NF-PSF/PANI 0.05–0.5–4	>99.99	90	99	92	25	13	96
NF-PSF/PANI 0.1–0.5–4	>99.99	90	99	92	25	25	96
NF-PSF/PANI 0.1–0.5–1	>99.99	69	73	45	23	4	69

**Table 9:** The Mg^2+^/Li^+^ separation factor of the developed TFC NF membranes.

Membrane Code	S_Mg/Li_
NF-PSF–2	9
NF-PSF/PANI 0.05–0.5–2	37
NF-PSF–4	8
NF-PSF/PANI 0.1–0.5–4	58

**Table 10:** MWCO and average pore size of the developed TFC NF membranes.

Membrane Code	MWCO, Da	Average Pore Size, nm
NF-PSF–2	300	310
NF-PSF/PANI 0.05–0.5–2	260	307
NF-PSF–4	300	326
NF-PSF/PANI 0.1–0.5–4	240	305

**Table 11:** Membrane performance of the developed TFC NF membranes after chemical treatment.

Membrane Code	Water Permeation (ΔP = 0.5 MPa), L·m^−2^h^−1^			Rejection of MgSO_4_, %		
	Before	After Chlorine Treatment	After Acid Treatment	Before	After Chlorine Treatment	After Acid Treatment
NF-PSF–2	64	65	67	>99.99	99	98
NF-PSF/PANI 0.05–0.5–2	64	64	64	>99.99	>99.99	>99.99
NF-PSF–4	21	23	25	>99.99	99	99
NF-PSF/PANI 0.1–0.5–4	40	40	40	>99.99	>99.99	>99.99

**Table 12:** Comparison of nanofiltration performance of the developed TFC NF membranes with membranes with an interlayer reported in the literature.

**Composition**			**Membrane** **Permeance, L·m^−2^·h^−1^·bar^−1^**	**R (MgSO_4_), %**	**R (Na_2_SO_4_), %**	**S (Mg^2+^/Li^+^)**	**Ref.**
**Membrane Support**	**Interlayer**	**PA Layer**					
PSF	PDA/PEI	PIP/TMC	7.5	95	97	-	[53]
PSF	TA	PEI/TMC	1.7	95	72	22	[65]
	TA-Cu		2.9	97	76	26.5	
PSF	PEI	TMC/DETA	3.2	94	-	11	[66]
PES	COF-A	MPD + PIP/ Tp + TMC	16.3	98	98	40.4	[38]
PES	Hyaluronic acid	PIP/TMC	29.5	91	95	-	[67]
PES	PDADMAC+ carboxylated cellulose nanocrystal	PEI/TMC	3.4	-	-	5.8	[68]
PES/PVP/PEG 400	GO-hyperbranched polyamide-amine	PIP/TMC	14.3	88	97	-	[69]
PSF/PVP K30	PANI	PIP/TMC	12.8	>99.99	84	37	This work
			8	>99.99	90	58	

## Data Availability

The data are contained within the manuscript.

## Supplementary Materials

The following supporting information can be downloaded at: https://www.mdpi.com/article/10.3390/polym17091199/s1. Figure S1: The scheme of oxidative polymerization of aniline. Figure S2: FTIR-spectra of reference UF-0 and UF PSF/PANI membranes. Figure S3: Water permeation (J_water_) of TFC NF membranes with a PANI intermediate layer depending on the concentration of monomers in the IP reaction (ΔP = 0.5 MPa).
