# Supplementary material for: Correlation Between Conditions of Polyaniline Interlayer Formation and the Structure and Performance of Thin-Film Composite Membranes for Nanofiltration Prepared via Interfacial Polymerization
Source: Polymers (Basel). 2025 Apr 28;17(9):1199. doi: 10.3390/polym17091199 (PMC12073387; doi:10.3390/polym17091199)
Supplement: Supplementary file 1 [file polymers-17-01199-s001.zip › polymers-3578447-supplementary.pdf]

Supporting Information

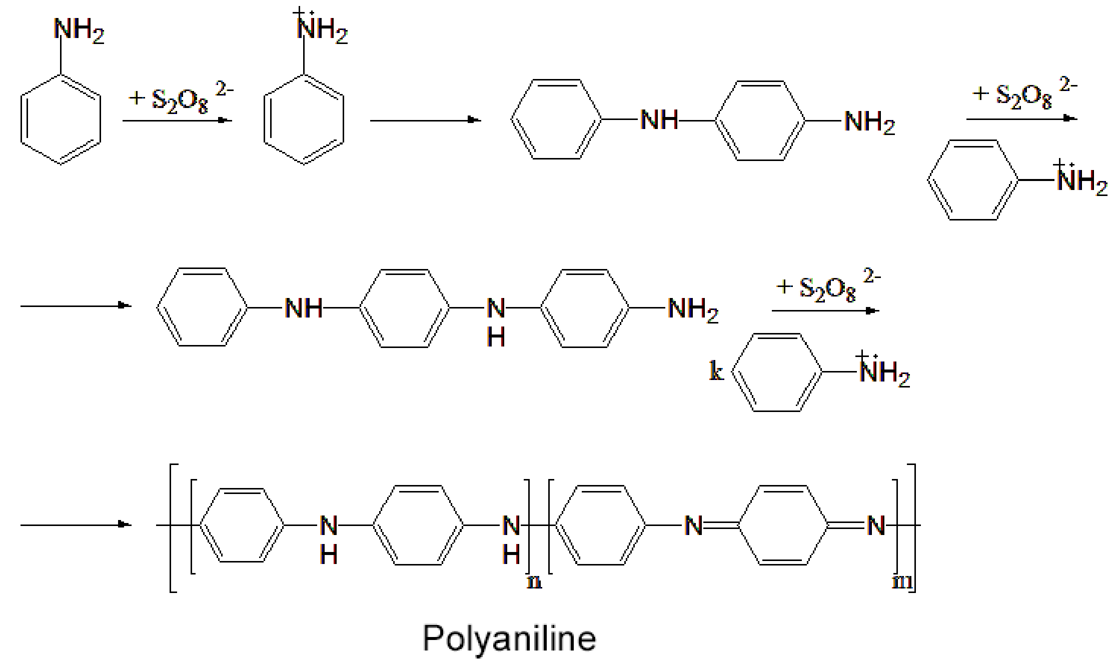

Figure S1. The scheme of oxidative polymerization of aniline

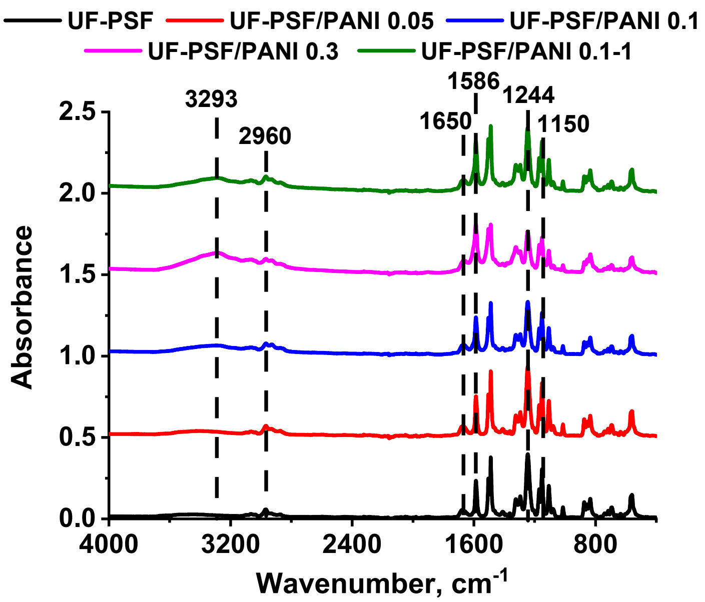

Figure S2. FTIR-spectra of reference UF-0 and UF PSF/PANI membranes.

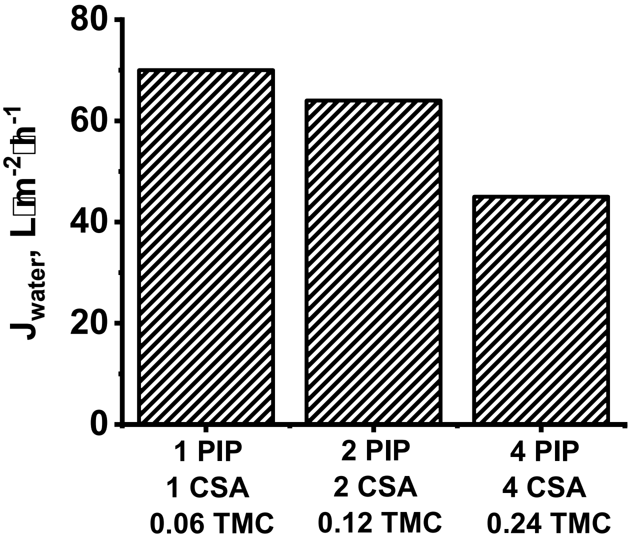

Figure S3. Water permeation (J_{water}) of TFC NF membranes with PANI intermediate layer depending on the concentration of monomers in the IP reaction (ΔP = 0.5 MPa)
